# Production and monitoring of biomass and fucoxanthin with brown microalgae under outdoor conditions

**DOI:** 10.1002/bit.27657

**Published:** 2020-12-31

**Authors:** Fengzheng Gao, Marta Sá, Iago Teles (Cabanelas, ITD), René H. Wijffels, Maria J. Barbosa

**Affiliations:** ^1^ Agrotechnology and Food Sciences, Bioprocess Engineering, AlgaePARC Wageningen University Wageningen The Netherlands; ^2^ Aquaculture, Faculty Biosciences and Aquaculture Nord University Bodø Norway

**Keywords:** chemometric modelling, fluorescence spectroscopy, fucoxanthin, *Phaeodactylum tricornutum*, *Tisochrysis lutea*

## Abstract

The effect of light on biomass and fucoxanthin (Fx) productivities was studied in two microalgae, *Tisochrysis lutea* and *Phaeodactylum tricornutum*. High and low biomass concentrations (1.1 and 0.4 g L^−1^) were tested in outdoor pilot‐scale flat‐panel photobioreactors at semi‐continuous cultivation mode. Fluorescence spectroscopy coupled with chemometric modeling was used to develop prediction models for Fx content and for biomass concentration to be applied for both microalgae species. Prediction models showed high *R* ^2^ for cell concentration (.93) and Fx content (.77). Biomass productivity was lower for high biomass concentration than low biomass concentration, for both microalgae (1.1 g L^−1^: 75.66 and 98.14 mg L^−1^ d^−1^, for *T. lutea* and *P. tricornutum*, respectively; 0.4 g L^−1^: 129.9 and 158.47 mg L^−1^ d^−1^, *T. lutea* and *P. tricornutum*). The same trend was observed in Fx productivity (1.1 g L^−1^: 1.14 and 1.41 mg L^−1^ d^−1^, *T. lutea* and *P. tricornutum*; 0.4 g L^−1^: 2.09 and 1.73 mg L^−1^ d^−1^, *T. lutea* and *P. tricornutum*). These results show that biomass and Fx productivities can be set by controlling biomass concentration under outdoor conditions and can be predicted using fluorescence spectroscopy. This monitoring tool opens new possibilities for online process control and optimization.

## Graphical abstract

Biomass and fucoxanthin outdoor production and monitoring with brown microalgae

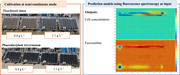

## 1 INTRODUCTION

Fucoxanthin (Fx) is the main carotenoid in marine brown algae, playing a key role in photosynthesis (Miyashita et al., 2011; Wang et al., 2019). Recently, Fx has gained high attention due to its biological properties, such as antioxidant, antiobesity, and antidiabetic, possible to be used in cosmetics, pharmaceuticals, and nutraceuticals (Fung et al., 2013; Guedes et al., 2011; Maeda et al., 2018). At the moment, edible brown seaweed are used as feedstock for industrial Fx production, despite their low concentration in Fx (0.01–3.7 mg g^−1^ dry weight [DW]) when comparing with other sources, such as microalgae (2.24–26.6 mg g^−1^ DW; Kim et al., 2012b; Li et al., 2019; Lu et al., 2018; Petrushkina et al., 2017; Terasaki et al., 2009; Wang et al., 2018).

Microalgae are considered sustainable feedstocks for food, feed and chemical ingredients. Several microalgae have been studied for their capacity to accumulate high contents of Fx, such as *Tisochrysis lutea* and *Phaeodactylum tricornutum* (Gao et al., 2020b). It is reported in literature that *T. lutea* can accumulate up to 1.82% DW of Fx (Kim et al., 2012b; Mohamadnia et al., 2020), while *P. tricornutum* can accumulate as much as 5.92% DW (McClure et al., 2018). Therefore, both microalgae are promising candidates for Fx production at industrial scale. Although many studies have been done related to Fx production in microalgae at indoor conditions, few studies were performed under outdoor conditions with fluctuating light intensities, which has a direct impact on biomass and bioproducts. Therefore, this study intends to fill that gap, by investigating the effect of different incident light intensities on biomass and Fx productivity in *T. lutea* and *P. tricornutum*.

In line with the efforts being done to improve microalgae industrial production, there is the need for a reliable online monitoring tool to allow better process control and understanding. Nowadays, most biological parameters monitored in microalgae cultivation are based in off‐line measurements, where a sample is withdrawn from the cultivation system, and later analyzed (Acién Fernández et al., 1998, 2000). Some of these analyses, like Fx content measured by HPLC, are laborious and time‐consuming, losing the opportunity to control their content online and to harvest the cultures when higher yields are achieved. Within the spectrophotometric technologies, fluorescence spectroscopy has been reported as a suitable technique for monitoring several compounds, simultaneously and at real‐time, noninvasively and nondestructively, in microalgae production (Kondo et al., 2017; Lavine & Workman, 2005; Sá et al., 2019b, 2020a, 2020b). However, until now, the prediction models were used for a specific microalga only, giving no guarantee that the same model could be used for the prediction of the same parameter in different microalgae.

In this work, *T. lutea* and *P. tricornutum* were cultivated in pilot‐scale outdoors flat‐panel photobioreactors (PBRs), at different cell concentrations, to evaluate the effect of specific light intensity on biomass and Fx productivities. Reactors were run in parallel, such that the light intensity at the surface of each reactor was the same. Two prediction models were built for cell concentration and for Fx content, coupling fluorescence spectra acquired from both microalgae. The spectral regions with higher relevance for the prediction models were also studied, providing a better understanding of how fluorescence can be useful to monitor these parameters. This work provides a new approach to monitor microalgal growth and composition using online fluorescence.

## 2 MATERIAL AND METHODS

### 2.1 Preculture conditions and scale‐up procedure

*T. lutea* and *P. tricornutum* strains were provided by NECTON, S.A. Precultures of both microalgae were incubated in 2 L bottles with 1.8 L of culture, at 27°C, with an incident light of 100 µmol m^−2^ s^−1^, day:night cycle (18:6 h), and bubbled with air enriched with 2% CO_2_. The cultivation medium is filtered natural sea water (Sartoguard®; PES membrane, 0.1 µm pore size; Sartorius), collected from the North Sea (The Netherlands), supplemented with 40 mM of NaNO_3_ and 2 ml L^−1^ of micronutrients solution (NutriBloom plus; Phytobloom®): 4 mM NaNO_3_, 0.2 mM KH_2_PO_4_, 0.05 M ethylenediaminetetraacetic acid, 0.04 mM FeCl_3_.6H_2_O, 2 µM ZnCl_2_, 2 µM ZnSO_4_, 2 µM MnCL_2_.2H_2_O, 0.2 µM Na_2_MoO_4_.2H_2_O, 0.2 µM CoCl_2_.6H_2_O, 0.2 µM CuSO_4_.5H_2_O, 4 µM MgSO_4_.7H_2_O, 0.07 mg L^−1^ Tiamine, 0.01 mg L^−1^ Biotin, 0.006 mg L^−1^ B_12_. The pH of the medium was adjusted to 8.0 containing 20 mM HEPES.

In the first scale‐up step, three 2 L bottles were used to inoculate one 25 L flat‐panel (Algae‐Germ) with an initial optical density at 750 nm (optical density [OD]_750_) of 0.2, approximately, with the same medium composition (filtered natural sea water, 40 mM of NaNO_3_, and 2 ml L^−1^ of NutriBloom plus). The culture was mixed by enriched air, with 2% CO_2_, under continuous illumination provided by white fluorescent lamps (250 µmol m^−2^ s^−1^). The temperature was controlled by a heating‐cooling system at 30°C and 20°C, for *T. lutea* and *P. tricornutum*, respectively.

### 2.2 PBRs setup and outdoor operation conditions

The outdoor experiments were performed between 12 and 19 of September 2019 for *T. lutea*, and between 4 and 16 of October for *P. tricornutum*. Four 40 L flat‐panel PBRs (Green Wall Panel®‐III; F&M Fotosintetica & Microbiologica S.r.l.; Rodolfi et al., 2017) were inoculated with culture from the 25 L flat‐panel PBR described previously, to a maximum cultivation volume of 38 L (initial OD_750_ of 0.2, approximately). The PBRs were firstly run at batch mode until they reach the concentrations under study: 0.4 g L^−1^ DW biomass for low biomass concentration level (*T. lutea* OD_750_ of 1; *P. tricornutum* OD_750_ of 0.7), and 1.1 g L^−1^ DW biomass for high biomass concentration level (*T. lutea* OD_750_ of 3; *P. tricornutum* OD_750_ of 2). Every morning (at 9:30 a.m.) the cultures were diluted with filtered natural sea water to 0.4 and 1.1 g L^−1^. NutriBloom plus and sterile NaNO_3_ solution were added to maintain the same micronutrients and nitrogen concentrations.

The 40 L flat‐panel PBRs were operated under controlled temperature, by a heating‐cooling system, between 27°C and 30°C for *T. lutea*, and 20°C and 22°C for *P. tricornutum*. The daily sunlight radiation was measured by Li‐COR Quantum, model Q 47183, each minute. The daily total irradiance (mol m^−2^ d^−1^) was obtained from the sum of all the recorded light intensity (µmol m^−2^ s^−1^) each cultivation day. The medium used in the outdoor experiments was the same as described previously. The culture was mixed by filtered air. The pH was set to 8.0 for *T. lutea* and 7.5 for *P. tricornutum*, and controlled by CO_2_ injection.

### 2.3 Sampling and DW measurement

A sample (~400 ml) was collected from each flat panel PBR to measure biomass concentration, Fx content and fluorescence spectroscopy. *T. lutea* and *P. tricornutum* growth was assessed by OD_750_ and DW. The OD was measured using a DR 5000™ UV‐Vis Laboratory Spectrophotometer (Hach) in duplicate. DW was measured according to a method described previously (Gao et al., 2020a).

### 2.4 Fx extraction and measurement

Fx quantification was performed using freeze‐dried biomass according to the method reported previously (Gao et al., 2020b). The biomass, around 2 mg, was extracted four times with 100% ethanol, in bead beating tubes (Lysing Matrix E, 2 ml; MP Biomedicals). The supernatants were collected, dried under N_2_ stream, and resuspended with 3 ml of 100% methanol. The sample was filtered through a syringe filter (SPARTAN® RC 30, 0.2 µm pore size; Whatman®) to a HPLC amber vial, and analyzed in an UPLC Shimadzu Nexera X2, equipped with a quaternary pump and DAD, and Kinetex C18 column (5 μm, 100 Å, 150 × 4.6 mm). The injection volume was 20 µl. The elution solvents were (A) 0.5 M ammonium acetate in methanol:milliQ water (85:15), (B) acetonitrile:milliQ water (90:10), and (C) 100% ethylacetate. Each run takes 53 min, with the following elution program (time in minutes: solvent concentration in %): 5 min:A(60)B(40)C(0), 10 min:A(0)B(100)C(0), 40 min:A(0)B(30)C(70), 45 min:A(0)B(30)C(70), 46 min:A(0)B(0)C(100), 47 min:A(0)B(100)C(0), 48 min:A(60)B(40)C(0), 53 min:A(60)B(40)C(0). A calibration curve (0, 2, 4, 6, 8, 10 μg ml^−1^) was prepared with Fx standard (Sigma‐Aldrich) in 100% methanol.

### 2.5 Data analysis and calculations

Experimental results were expressed as mean value ± *SD*. Differences between groups were tested for significance by the least significant difference mean comparison, using the IBM® SPSS® Statistics software (version 25). The relationship between variables was determined by one‐way analysis of variance at a significance level of 0.05 using a Duncan post hoc test.

The daily dilution rate (D; d^−1^) was calculated using Equation (1);

(1) $$D=(1-Cx_{0}/Cx_{t})/(t-t_{0})$$

where *Cx*_t_ is the measured biomass concentration (g L^−1^), and *Cx*_0_ is the setting biomass concentration, that is, 0.4 g L^−1^ and 1.1 g L^−1^ for LBC and HBC, respectively; t − t_0_ = 1 d.

The volumetric biomass productivity (*P*_*X*_; g L^−1^ d^−1^) was calculated using Equation (2);

(2) $$P_{X}=Cx_{t}\times D$$

where *Cx*_t_ is the measured biomass concentration (g L^−1^), and D is the dilution rate (d^−1^).

The volumetric Fx productivity (*P*_Fx_; mg L^−1^ d^−1^) was calculated using Equation (3);

(3) $$P_{Fx}=C_{Fx}\times P_{X}$$

where *C*_Fx_ is the Fx concentration (mg g^−1^), and *P*_*X*_ is the volumetric biomass productivity (g L^−1^ d^−1^).

### 2.6 Fluorescence spectroscopy

Excitation‐emission matrices (EEMs) were acquired in an excitation range between 250 and 790 nm, and emission range between 260 and 800 nm, both in 5 nm steps (Shimadzu RF‐6000). The analysis was performed in a quartz cuvette of 3 ml, with samples collected directly from the PBRs (no dilution), and no cell sedimentation was observed during the measurement. Excitation and emission monochromator slit widths were 3 nm, with a scan speed of 12,000 nm/minute.

### 2.7 Prediction models development

The EEMs were preprocessed by removal of the Rayleigh scatter and inner filter effect. The first order Rayleigh scatter was replaced by empty values, and the second order was replaced with an interpolation of surrounding data points (Bahram et al., 2006). Inner filter effects can occur by excessive biomass concentration, and that was also corrected by the algorithm whenever present. Fluorescence signals corresponding to emission wavelengths (y‐axis) shorter than the excitation wavelengths (x‐axis) were replaced by zeros.

The preprocessed EEMs were correlated with cell concentration and Fx content (predicted variables) using the multiway version of Projection to Latent Structures (PLS), N‐PLS (Bro, 1996). The predicted variables were converted into their logarithm base 10, to normalize their distribution and facilitate the modelling task.

Three different datasets were studied: first, using data only from the *T. lutea* experiments; second, using data only from the *P. tricornutum* experiments; third, using combined data of both *T. lutea* and *P. tricornutum* experiments.

For all modelling strategies, the datasets were split randomly into a training and validation sets, corresponding to 75% and 25% of the total data, respectively. The training set was used to calibrate the model and to optimize the number of latent variables (LVs), by leave‐one‐out cross‐validation (LOOCV). Shortly, in LOOCV, one sample is removed from the training set, the PLS model is built with the remaining samples, and the quality is assessed; this procedure was repeated until all the samples in the training set were left‐out once. The validation set was used to validate the model, with the optimal number of LVs, defined by the LOOCV. The process was repeated four times, validated by a fourfold double cross‐validation, until every sample was used in the external validation set once (Filzmoser et al., 2009).

For the third dataset only (*T. lutea* and *P. tricornutum* data compiled together), an additional modelling strategy was studied. First, the data from the *T. lutea* experiments was used as training dataset, and the *P. tricornutum* experiments as validation set; then, the inverse was tested, using *P. tricornutum* experiments as training set and *T. lutea* experiments as validation set. This strategy enables to understand the prediction robustness of the predicted variables when a model was developed based in the dataset of a particular microalga.

To verify the models' quality, several parameters were evaluated, such as the variance explained in the predicted variables (%), the root mean square error of cross‐validation (RMSECV) and prediction (RMSEP), and the *R* ^2^ and slopes of the training and external validation sets.

After assessing the quality of the models, a final model was built for each predicted variable, using the whole dataset and the optimal number of LVs defined previously. By this way it is possible to determine and examine the regression coefficient values, and therefore evaluate the weight of each excitation‐emission pair for the prediction model.

All analyses were performed using the drEEM toolbox (http://www.models.life.ku.dk/dreem) and n‐way toolbox in MATLAB (The MathWorks®; Andersson & Bro, 2000).

## 3 RESULTS AND DISCUSSION

### 3.1 Biomass content and productivity

In outdoor microalgae production, the biomass is exposed to a continuously changing light intensity which cannot be changed manually. However, the biomass concentration can be controlled, to result in different quantities of light received per cell. Two biomass concentrations, 0.4 g L^−1^ (low biomass concentration) and 1.1 g L^−1^ (high biomass concentration), were tested at outdoor conditions, to investigate the effect of light on microalgal growth. Initially, a third level of biomass concentration was tested with *T. lutea* (1.9 g L^−1^), but no growth was observed due to the light limitation. Therefore, the studies proceed with the two concentration' levels mentioned.

**Figure 1:**
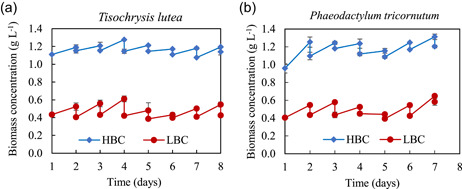
Growth of *Tisochrysis lutea* (a) and *Phaeodactylum tricornutum* (b) at semi‐continuous mode. Two cell concentrations were studied: low biomass concentration (LBC) of 0.4 g L^−1^ DW (circles, red), and high biomass concentration (HBC) of 1.1 g L^−1^ DW (diamond, blue) [Color figure can be viewed at wileyonlinelibrary.com]

Biomass concentrations were kept constant during the semi‐continuous growth (Figure 1). The specific growth rates of both microalgae were higher at 0.4 g L^−1^ than 1.1 g L^−1^ (Table 1). The average growth rate for low biomass concentration was 3.8‐ and 3.5‐fold higher than at high biomass concentration in *T. lutea* and *P. tricornutum*, respectively. This difference can be explained by the photolimitation effect (Acién Fernández et al., 2000). Due to the high biomass concentration, no light was detected on the back of the flat‐panel PBRs. Also, an increase in cell density decreases the light received by the culture due to self‐shading (Gómez‐Loredo et al., 2016). This low light penetration and low distribution resulted in a lower growth rate. This phenomenon was less intense at 0.4 g L^−1^, except for days with low light intensity (e.g., rainy and/or foggy days, such as Day 6 in *T. lutea* cultivation, Days 4 and 5 in *P. tricornutum* cultivation).

**Table 1:** Biomass (specific growth rate and productivity) and fucoxanthin (content and productivity) from *Tisochrysis lutea* and *Phaeodactylum tricornutum* experiments

			*Tisochrysis lutea*		*Phaeodactylum tricornutum*	
			LBC	HBC	LBC	HBC
Mean of the total daily light (mol m^−2^ d^−1^)			18.54 ± 7.76		9.58 ± 5.10	
Biomass	Specific growth rate (d^−1^)	Average	0.23 ± 0.09	0.06 ± 0.03	0.28 ± 0.10	0.08 ± 0.04
		Minimun	0.07	0.04	0.10	0.03
		Maximun	0.36	0.12	0.42	0.15
	Productivity (mg L^−1^ d^−1^)	Average	129.9	75.66	158.47	98.14
Fucoxanthin	Content (% DW)	Average	1.37 ± 0.12	1.78 ± 0.12	1.09 ± 0.12	1.33 ± 0.18
		Minimun	1.25	1.6	0.93	1.2
		Maximun	1.56	2.03	1.3	1.58
	Productivity (mg L^−1^ d^−1^)	Average	2.09 ± 0.89	1.14 ± 0.47	1.73 ± 0.73	1.41 ± 1.01

*Note*: Two cell concentrations were studied: LBC of 0.4 g L^−1^ DW, and HBC of 1.1 g L^−1^ DW.

Abbreviations: DW, dry weight; HBC, high biomass concentration; LBC, low biomass concentration.

**Figure 2:**
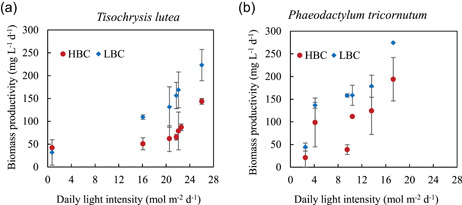
Biomass productivity (mg  L^−1^ d^−1^) of *Tisochrysis lutea* (a) and *Phaeodactylum tricornutum* (b). Two cell concentrations were studied: low biomass concentration (LBC) of 0.4 g L^−1^ DW (diamond, blue), and high biomass concentration (HBC) of 1.1 g L^−1^ DW (circles, red) [Color figure can be viewed at wileyonlinelibrary.com]

In *T. lutea*, the biomass productivity ranged between 31.78 and 223.12 mg L^−1^ d^−1^ in low biomass concentration experiments, and 41.98–143.71 mg L^−1^ d^−1^ in high biomass concentration (Figure 2a). Although the minimum productivity value was similar in both biomass concentrations, the maximum productivity was almost two times higher at low biomass concentration. This result was also observed by Ippoliti et al. (2016b), which concluded that higher biomass productivities can be reached by operating at higher dilution rates, due to a decrease in the light‐limitation effect. It was reported that *T. lutea* biomass maximum productivity can reach 200–300 mg L^−1^ d^−1^, in tubular PBRs (3.0 m^3^ capacity) at outdoor conditions in Almería, Spain (Ippoliti et al., 2016a; 2016b), which is slightly higher than value measured in the low biomass concentration experiment. Also, an average value of 75 mg L^−1^ d^−1^ was reported, which is similar to the average productivity observed in the high biomass concentration experiments (75.66 mg L^−1^ d^−1^), and lower than in low biomass concentration experiments (129.9 mg L^−1^ d^−1^; Table 1; van Bergeijk et al., 2010). A lower average biomass productivity (40 mg L^−1^ d^−1^) was reported for *Isochrysis zhangjiangensis* cultivated in 10 m^2^ floating PBRs, in an offshore test field in Lingshui Bay, in Dalian, China (38°87′N, 121°55′E; Zhu et al., 2019).

In *P. tricornutum* experiments, the biomass productivity ranged between 21.15 and 274.27 mg L^−1^ d^−1^ in low biomass concentration, and 44.41–194.07 mg L^−1^ d^−1^ in high biomass concentration (Figure 2b). Similar to *T. lutea* experiments, the minimum productivity of *P. tricornutum* was closer for both concentrations, whereas the maximum productivity was higher in low biomass concentration. The results obtained in this work were similar to those reported by Steinrücken et al. (2018). The same cultivation system was used, to study three different *P. tricornutum* strains, under Norwegian climate conditions. During spring, when the light intensity was higher (39 mol m^−2^ d^−1^), the productivity ranged between 250 and 300 mg L^−1^ d^−1^. However, when the light intensity decreased to 20 mol m^−2^ d^−1^ (summer and autumn), the biomass productivity decreased too (90–160 mg L^−1^ d^−1^; Steinrücken et al., 2018). In the present study, although *P. tricornutum* was cultivated at a lower average light intensity of 9.58 mol m^−2^ d^−1^, the average biomass productivity was similar to their results obtained at 20 mol m^−2^ d^−1^ (158.47 mg L^−1^ d^−1^ at 0.4 g L^−1^, and 98.14 mg L^−1^ d^−1^ at 1.1 g L^−1^; Table 1). Moreover, lower biomass productivities (29–79 mg L^−1^ d^−1^) were reported when cultivated in 270 vertical prototype‐outdoor column PBRs (270 L, column height 220 cm, diameter 55 cm), at semi‐continuous mode in January and February in Italy (Simonazzi et al., 2019). Higher biomass productivity, 1400 mg L^−1^ d^−1^, was achieved in southern latitudes in Spain, with higher mean daily irradiance (1100 μmol m^−2^ s^−1^; Acién Fernández et al., 2003). In our study, the light intensity was low (Figure S1), with mean daily irradiance approximately 200 μmol m^−2^ s^−1^.

In both *T. lutea* and *P. tricornutum* experiments, low biomass concentration resulted in higher average growth rate and biomass productivity. Both growth rate and biomass productivity were positively correlated with the light intensity.

### 3.2 Fx content and productivity

It is known that an increase in Fx content, a light‐harvesting pigment, is stimulated by the self‐shading effect of cells or low light intensity, to absorb sufficient light for photosynthesis (Faraloni & Torzillo, 2017). This phenomenon was also observed in this work, where a higher self‐shading effect in high biomass concentration led to a decrease in the received light per cell, hence increasing the Fx content. Also, when the light intensity was higher, the Fx content decreased (Table 1). In *T. lutea*, high biomass concentration had 1.3‐fold more Fx than low biomass concentration, while in *P. tricornutum* that difference was smaller (1.2‐fold). On average, the Fx content in *T. lutea* was higher than in *P. tricornutum*, for both biomass concentrations (Table 1 and Figure S2).

The Fx reported in literature range between 0.22% and 1.82% for *T. lutea*, and from 0.19% to 5.92% in *P. tricornutum* (Gao et al., 2020b; Ishika et al., 2017; Kim et al., 2012a; 2012b; McClure et al., 2018; Mohamadnia et al., 2020; Wu et al., 2016). The results obtained in this work are in accordance to what other authors reported for the same growth conditions, where low *P. tricornutum* biomass (0.24–0.36 g L^−1^) led to a lower Fx content (0.21%–0.55% DW). For *T. lutea*, a Fx content of 1.82% was reported before (Kim et al., 2012b), similar to the value reached in the present study.

**Figure 3:**
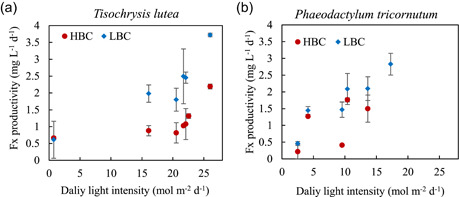
Fucoxanthin productivity (mg L^−1^ d^−1^) of *Tisochrysis lutea* (a) and *Phaeodactylum tricornutum* (b). Two cell concentrations were studied: low biomass concentration (LBC) of 0.4 g L^−1^ DW (diamond, blue), and high biomass concentration (HBC) of 1.1 g L^−1^ DW (circles, red) [Color figure can be viewed at wileyonlinelibrary.com]

In our experiments with controlled temperature, light was the major parameter that affected biomass and Fx productivities. Fx productivity was directly proportional to the daily light intensity (Figure 3a,b). As observed before, the Fx productivity trend was positively related with daily light intensity (Figure 3). Although large *SD*s were observed in the average Fx productivities due to the drastic daily radiation fluctuation, the values were higher in low biomass concentration, for both microalgae (Figure 3 and Table 1). The Fx productivities found in this work are comparable to what was described previously in the literature for indoor experiments. A screening of sixteen *T. lutea* strains revealed an average productivity between 1.07 and 5.28 mg L^−1^ d^−1^, with a maximum value of 7.96 mg L^−1^ d^−1^, under continuous low light intensity (approximately 5 mol m^−2^ d^−1^; Sun et al., 2019). Similar values were reported for *P. tricornutum*, where light intensities ranging between 4 and 9 mol m^−2^ d^−1^ resulted in 1.05–1.10 mg L^−1^ d^−1^ (Aslanbay Guler et al., 2019), compared to 1.2–1.4 mg L^−1^ d^−1^ observed in this work. However, a higher value (4.73 mg L^−1^ d^−1^) was reported when cultivating *P. tricornutum* at higher light intensity (approximately 26 mol m^−2^ d^−1^; Gao et al., 2017).

Fx content and productivity proved to be sensitive to light intensity variations on a daily basis. This is an important parameter to consider when aiming for harvesting biomass with the higher content possible.

### 3.3 Prediction models

Fluorescence spectra were acquired from all samples to develop prediction models for two parameters: biomass concentration and Fx content. The quality of those prediction models was evaluated using several modelling strategies, but always with fluorescence data as single input. Only after determining the optimal LVs number, a final prediction model can be created in a function of a matrix (regression coefficient matrices), where the spectra regions with positive/negative contribution could be evaluated.

#### 3.3.1 Cell concentration prediction models

Cell concentration prediction models, based on DW, were performed in three different scenarios: using data from *T. lutea* only, using data from *P. tricornutum* only, and using the combination of both datasets.

The results of the cell concentration prediction models, for both microalgae individually, showed to be robust, with *R* ^2^ above .95 and slopes close to 1, which indicates that the same trend is observed between observed and predicted values (Table 2). The RMSEP, that represents the average distance between the observed data points and the predicted ones, were low for both prediction models. In the model developed with *T. lutea* data, the RMSECV is close to the RMSEP, which indicates the absence of over‐fitting. A higher difference was found for the model developed with *P. tricornutum* data. It is also worth noting that the explained variance for the *T. lutea* model is slightly lower than for the *P. tricornutum* model. This is due to the amount of data points used to build the prediction models. For the *P. tricornutum* model, more data points were collected, since it included the initial period of batch mode (where the biomass grew until the desired cell concentration was reached, data not shown), and not only the phase of the semi‐continuous experiments (as done with *T. lutea*), thus more variability of cell concentrations was included.

**Table 2:** Performance parameters of cell concentration models

	Training		Validation		RMSE (log_10_ g L^−1^)		Variance (%)
	*R* ^2^	Slope	*R* ^2^	Slope	CV	*p*	
*T. lutea*	0.98 ± .01	0.95 ± 0.03	0.96 ± .03	0.97 ± 0.11	0.037 ± 0.012	.035 ± .008	97.10 ± 1.98
*P. tricornutum*	0.97 ± .01	0.94 ± 0.05	0.96 ± .01	0.97 ± 0.12	0.052 ± 0.008	.043 ± .004	99.16 ± 0.23
*T. lutea + P. tricornutum*	0.96 ± .01	0.98 ± 0.06	0.93 ± .05	0.99 ± 0.15	0.052 ± 0.013	.048 ± .008	98.08 ± 1.33

*Note*: Models were built using 75% of the data for training and 25% for validation. Three datasets where tested: using data only from *Tisochyrysis lutea* (*n* training = 21, *n* validation = 7); only from *Phaeodactylum tricornutum* (*n* training = 36, *n* validation = 12); and combined data of *T. lutea* and *P. tricornutum* (*n* training = 57, *n* validation = 19). The model performance parameters are: coefficients of determination (*R* ^2^) and slopes of linear regression for training and validation datasets, RMSE of CV and P, and captured variance.

Abbreviations: CV, cross‐validation; P, prediction; RMSE, root mean square errors.

**Figure 4:**
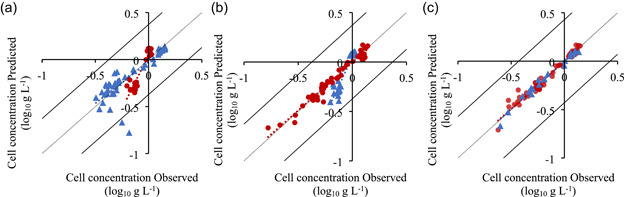
Cell concentration prediction models: (a) *Tisochrysis lutea* data for training (*n* = 28), and *Phaeodactylum tricornutum* data for validation (*n* = 48); (b) *P. tricornutum* data for training, and *T. lutea* data for validation; (c) combined data of *T. lutea* and *P. tricornutum*, random 75% for training (*n* = 57) and 25% for validation (*n* = 19; one of four partitions of training/validation datasets). Training (●, red) and validation (▲, blue) data in log_10_ g L^−1^ [Color figure can be viewed at wileyonlinelibrary.com]

The use of a technique such as fluorescence spectroscopy as a monitoring tool in microalgae cultivation is more interesting if the same model could be used to measure the same parameter in different microalgae. Therefore, data from *T. lutea* and *P. tricornutum* were compiled together, and new prediction models were developed. For this, three different validation strategies were tested: using *T. lutea* dataset (Figure 4a), using *P. tricornutum* dataset (Figure 4b), and a random validation using 25% of the total data (Figure 4c and Table 3). As it can be seen in the data distribution of Figure 4c, and in the model performance parameters in Table 3, compiling data from two different microalgae to predict cell concentration did not affect the overall prediction quality of the model. The *R* ^2^ and slope of the training and validation datasets were close to 1, the RMSEP was slightly higher, and the variance explained was 98%. However, it is possible to see from Figure 4a,b that, using cell concentration of one microalga to predict the cell concentration of the other, did not always result in a good prediction model. For instance, using *T. lutea* data to train the model revealed to be a poorer choice than using *P. tricornutum* data. Again, this might be caused by using data only from the semi‐continuous cultivation of *T. lutea*, with only two different cell concentrations. Therefore, to improve the prediction quality of the models it is suggested to use a wider range of cell concentrations for the training set.

**Table 3:** Performance parameters of fucoxanthin content models

	Training		Validation		RMSE (mg g^−1^)		Variance (%)
	*R* ^2^	Slope	*R* ^2^	Slope	CV	*p*	
*T. lutea*	0.84 ± .11	0.94 ± 0.07	0.63 ± .18	0.90 ± 0.13	0.038 ± 0.007	.031 ± .008	80.46 ± 12.24
*P. tricornutum*	0.83 ± .06	1.02 ± 0.08	0.64 ± .22	0.98 ± 0.17	0.084 ± 0.008	.067 ± .002	83.08 ± 8.46
*T. lutea* +* P. tricornutum*	0.91 ± .01	0.94 ± 0.05	0.77 ± .14	0.92 ± 0.10	0.071 ± 0.007	.053 ± .007	87.79 ± 4.83

*Note*: Models were built using 75% of the data for training and 25% for validation. Three datasets where tested: using data only from *Tisochrysis lutea* (*n* training = 21, *n* validation = 7); only from *Phaeodactylum tricornutum* (*n* training = 36, *n* validation = 12); and combined data of *T. lutea* and *P. tricornutum* (*n* training = 57, *n* validation = 19). The model performance parameters are: coefficients of determination (*R* ^2^) and slopes of linear regression for training and validation datasets, RMSE of CV and P, and captured variance.

Abbreviations: CV, cross‐validation; P, prediction; RMSE, root mean square errors.

Fluorescence spectroscopy has recently been studied to monitor cell concentration in other microalgae, such as *Dunaliella salina* and *Nannochloropsis oceanica* (Sá et al., 2017, 2019a, 2020a). Nevertheless, according to the authors knowledge, it is the first time that this technique is used to develop one prediction model able to be used in different microalgae species, revealing the high potential of this technique to be used in microalgae production.

#### 3.3.2 Fx content prediction models

Although the two microalgae in this study are different in many aspects (different classes, different morphologic characteristics and composition), both are reported in literature for its ability to produce and accumulate high amounts of Fx. Since pigment quantification is a laborious, expensive and time‐consuming methodology, the development of a prediction model able to be used online is of extreme importance for microalgae industrialization (Kondo et al., 2017).

**Figure 5:**
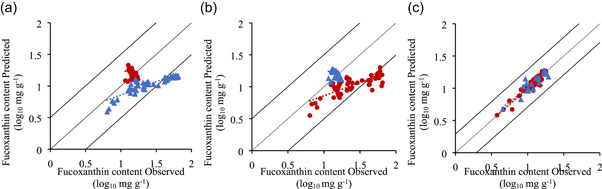
Fucoxanthin content prediction models: (a) *Tisochrysis lutea* data for training (*n* = 28), and *Phaeodactylum tricornutum* data for validation (*n* = 48); b) *P. tricornutum* for training, and *T. lutea* for validation; c) combined data of *T. lutea* and *P. tricornutum*, random 75% for training (*n* = 57) and 25% for validation (*n* = 19; one of four partitions of training/validation datasets). Training (●, red) and validation (▲, blue) data in log_10_ mg g^−1^ [Color figure can be viewed at wileyonlinelibrary.com]

A similar modeling approach as described previously was used. As before, more data points were collected during the *P. tricornutum* experiments, leading to a larger data distribution, with higher explained variance but also higher RMSEP and RMSECV, when comparing with *T. lutea* Fx prediction model (Table 3). For both microalgae, Fx prediction models had a higher *R* ^2^ for the training than the validation dataset, being legitime to consider a slight overfit of the prediction model, even with the use of an internal cross‐validation and external validation. This phenomenon was slightly mitigated when combining the two datasets (from *T. lutea* and *P. tricornutum*), and using 25% of data for the external validation (Figure 5c and Table 3). In this case, both *R* ^2^ were higher, as well as the variance explained. However, because of the lower variability found in Fx content in *T. lutea* experiments, and the presence of outliers in the *P. tricornutum* data distribution (data points plotted outside the line that represents two times the standard deviation), it is not advice to use data from one microalga to predict the pigment content of the other (Figure 5a,b). It is important to mention that the data distribution could be improved with acquisition of more data points, in a wider concentration range, to better train the model. Also, the sampling period could be adjusted to the middle or end of the day. When the cultures were sampled, daily at 9 a.m., they were exposed to only a few hours of sunlight, and not to the highest intensity of the day. It is reported in literature that chlorophyll content increases during the day, adapting to the light conditions, and decreases during the night (de Winter et al., 2013).

Previous works described the use of fluorescence spectroscopy to monitor chlorophylls and carotenoids, in different microalgae biomass, but not Fx (Sá et al., 2019b, 2020a). For those pigments, fluorescence spectroscopy had demonstrated already a high capacity to detect slight concentration differences, like the ones reported in this work.

#### 3.3.3 Regression coefficients of the final models for cell concentration and Fx

The performed experiments allowed the calibration of the prediction models for biomass concentration and Fx content for both microalgae. Fluorescence spectroscopy showed high accuracy in monitoring two parameters in two microalgae, simultaneously.

**Figure 6:**
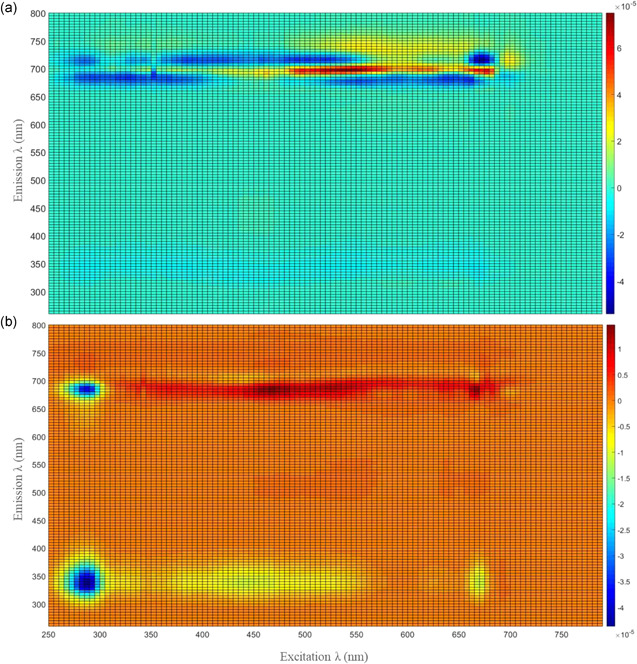
Regression coefficients of prediction models: (a) Cell concentration; (b) Fucoxanthin content. Training used 100% of the dataset. Excitation wavelengths in x‐axis, emission wavelengths in y‐axis, and fluorescence intensity scale bar on the right side [Color figure can be viewed at wileyonlinelibrary.com]

To be able to use the fluorescence spectroscopy as a monitoring tool in an industrial environment, a final model was created using the optimal number of LVs: 6 for biomass concentration, and 4 for Fx content. The regression coefficients matrix shows the weight of each excitation‐emission pair for each of the prediction models (Figure 6a,b).

In the cell concentration prediction model, it is possible to notice the importance of the emission wavelengths above 600 nm, negative or positively (Figure 6a). This region was described previously as the pigment emission band (Moberg et al., 2001; Sá et al., 2017, 2019b). In previous studies this region was already reported for its contribution in cell concentration prediction models (Sá et al., 2020a). However, in that study, it was also reported that the protein‐like region (excitation and emission wavelengths lower than 400 nm) also contributed for the cell concentration modelling, and that was not observed in the present work. On the contrary, for the Fx prediction model, both regions have a positive or negative contribution for the model (Figure 6b). Considering that most of the data was acquired from experiments where two different biomass concentration were used (0.4 and 1.1 g L^−1^), it is possible that this imposed variability may affected the Fx prediction models.

Using fluorescence spectroscopy as a monitoring tool for several components simultaneously has been reported (Lakowicz, 2006). Coupling with chemometric modelling, large amounts of information can be extracted from the EEMs, providing knowledge about the cultivation process at real‐time, revealing to be a powerful decision‐making tool.

## 4 CONCLUSIONS

This study investigated biomass and Fx productivities of *T. lutea* and *P. tricornutum* at pilot scale under outdoor conditions, and developed prediction models to monitor them. For both microalgae, high biomass concentration resulted in highest Fx content. However, low biomass concentration lead to higher biomass and Fx productivities. Prediction models were developed using fluorescence spectroscopy EEMs of both microalgae as only input. In general, high *R* ^2^ and explained variances were observed, with low RMSEs. Overall, it is possible to manipulate and monitor biomass and Fx contents using the same model per parameter, thus increasing industrial process control.

## CONFLICT OF INTERESTS

The authors declare that there are no conflict of interests.

## AUTHOR CONTRIBUTION

Fengzheng Gao contributed to investigation, methodology, formal analysis, data curation, software, visualization, writing—original draft, writing—review and editing. Marta Sá contributed to investigation, methodology, formal analysis, data curation, software, visualization, writing—original draft, writing—review and editing. Iago Teles (Cabanelas, ITD) contributed to project administration, formal analysis, methodology, supervision, writing—review and editing. René H. Wijffels contributed to project administration, supervision, writing—review and editing. Maria J. Barbosa contributed to project administration, formal analysis, funding acquisition, methodology, supervision, writing—review and editing. Fengzheng Gao and Marta Sá contributed equally to this work.

## Supporting information

Supporting information.

## Data Availability

The datasets generated and analysed during the current study will be available in the MAGNIFICENT Zenodo repository, after publication.
