# Supplementary material for: Production and monitoring of biomass and fucoxanthin with brown microalgae under outdoor conditions
Source: Biotechnol Bioeng. 2020 Dec 31;118(3):1355–65. doi: 10.1002/bit.27657 (PMC7986402; doi:10.1002/bit.27657)
Supplement: Supplementary file 1 — Supporting information. [file BIT-118-1355-s001.pdf]

Production and monitoring of biomass and fucoxanthin with brown microalgae under outdoor conditions

Fengzheng Gao[1,*,#], Marta Sá[1,#], Iago Teles (Cabanelas, ITD)[1], René H. Wijffels[1,2], Maria J. Barbosa[1]

1 Wageningen University, Bioprocess Engineering, AlgaePARC, P.O. Box 16, 6700 AA, Wageningen, Netherlands

2 Faculty Biosciences and Aquaculture, Nord University, N-8049 Bodø, Norway

* Corresponding author at: Wageningen University, Bioprocess Engineering, AlgaePARC, P.O. Box 16, 6700 AA, Wageningen, Netherlands

Fengzheng Gao, E-mail addresses: fengzheng.gao@wur.nl; gaofengzheng@outlook.com; Tel: +31317483595

[#] These authors contributed equally to this work.

Supporting information

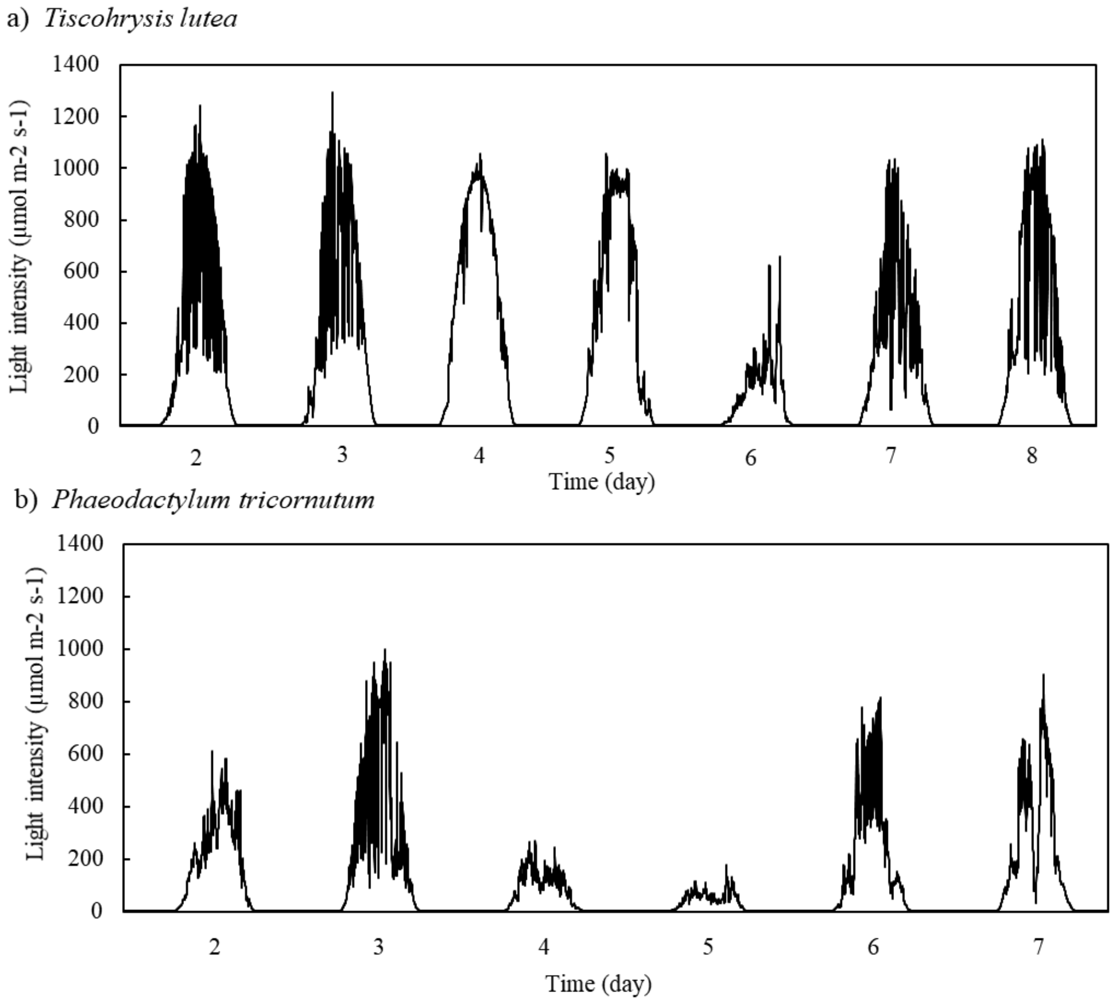

FIGURE S1 Real-time light intensity during the cultivation of *Tisochrysis lutea* (a) and *Phaeodactylum tricornutum* (b). Note: Light intensity was recorded each minute.

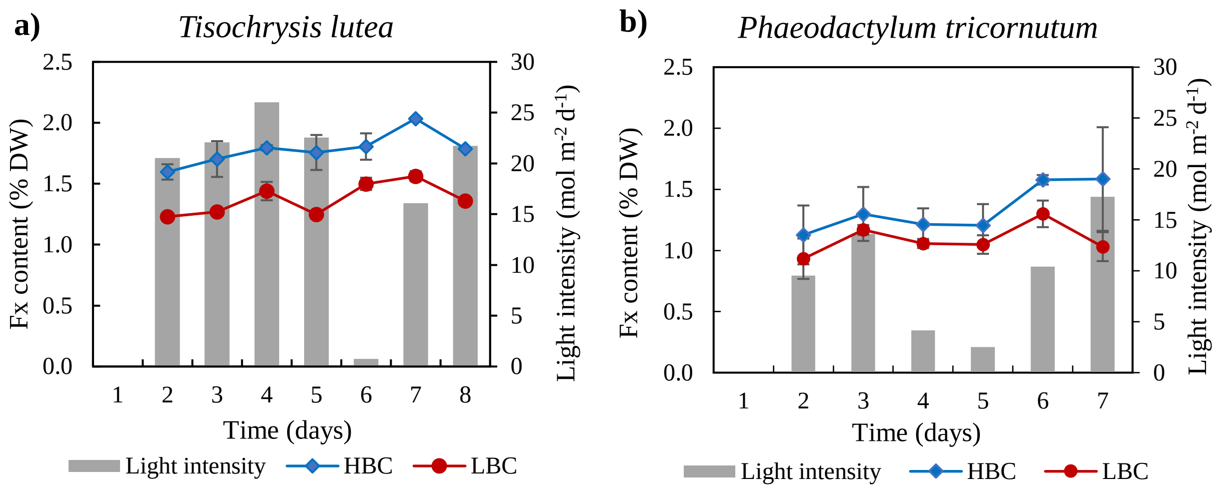

FIGURE S2 Fucoxanthin content (% DW) of *Tisochrysis lutea* (a) and *Phaeodactylum tricornutum* (b). Two cell concentrations were studied: low biomass concentration (LBC) of 0.4 g L^{-1} DW (diamond, blue), and high biomass concentration (HBC) of 1.1 g L^{-1} DW (circles, red)
